# Seroprevalence and Risk Factors of Brucella Infection in Dairy Animals in Urban and Rural Areas of Bihar and Assam, India

**DOI:** 10.3390/microorganisms9040783

**Published:** 2021-04-09

**Authors:** Ram Pratim Deka, Rajeswari Shome, Ian Dohoo, Ulf Magnusson, Delia Grace Randolph, Johanna F. Lindahl

**Affiliations:** 1 Department of Clinical Sciences, Swedish University of Agricultural Sciences (SLU), 750 07 Uppsala, Sweden; Ulf.Magnusson@slu.se (U.M.); J.Lindahl@cgiar.org (J.F.L.); 2 Department of Animal & Human Health, International Livestock Research Institute (ILRI), Nairobi 00100, Kenya; d.radolph@cgiar.org; 3 National Institute of Veterinary Epidemiology & Disease Informatics (NIVEDI), Bangalore 560064, India; rajeswarishome@gmail.com; 4 Professor Emeritus—Epidemiology, University of Prince Edward Island, Charlottetown, PE C1A 4P3, Canada; dohoo@upei.ca; 5 Food Safety System, Natural Resources Institute, University of Greenwich, Kent ME44TB, UK; 6 Department of Medical Biochemistry and Microbiology, Uppsala University, 751 05 Uppsala, Sweden

**Keywords:** brucellosis, epidemiology, dairy production, reproductive disease, risk factors

## Abstract

This study assessed seropositivity of Brucella infection in dairy animals and risk factors associated with it. The cross-sectional study used multi-stage, random sampling in the states of Bihar and Assam in India. In total, 740 dairy animals belonging to 534 households of 52 villages were covered under this study. Serological testing was conducted by indirect enzyme-linked immunosorbent assay (iELISA). Animal-level Brucella seropositivity was found to be 15.9% in Assam and 0.3% in Bihar. Seropositivity in urban areas (18.7%) of Assam was found to be higher than in rural areas (12.4%). Bihar was excluded from the risk factor analysis, as only one Brucella seropositive sample was detected in the state. A total of 30 variables were studied for assessing risk factors, of which 15 were selected for multivariable regression analyses following a systematic process. Finally, only three risk factors were identified as statistically significant. It was found that animals belonging to districts having smaller-sized herds were less likely (*p* < 0.001) to be Brucella seropositive than animals belonging to districts having larger-sized herds. Furthermore, the chance of being Brucella seropositive increased (*p* = 0.007) with the increase in age of dairy animals, but decreased (*p* = 0.072) with the adoption of artificial insemination (AI) for breeding. We speculated that the identified risk factors in Assam likely explained the reason behind lower Brucella seropositivity in Bihar, and therefore any future brucellosis control program should focus on addressing these risk factors.

## 1. Introduction

Globally, brucellosis is one of the most important zoonotic diseases of dairy cattle and buffaloes [1]. It is reported throughout the world; however, prevalence is often underreported in low- and middle-income countries [2]. The disease is considered endemic in India, and is being reported throughout the country. In sexually mature female cattle, the disease causes abortion, especially in the last trimester, retained placenta, and repeat breeding resulting in production losses [1,3]. Clinical manifestations of the disease in other bovine species like buffaloes, bison, and yak are similar to those in cattle [4]. The disease is caused by bacteria of the genus Brucella. There are diverse opinions in the scientific community in regards to presence of different species or sub-species of Brucella; however, it is generally accepted that there are at least twelve species of Brucella [5]. In cattle and buffalo, the infection is predominantly caused by *B. abortus* and *B. melitensis* [6].

Isolation and identification of Brucella organism is the gold standard for diagnosis, but the isolation is difficult and poses risk to human health because of the zoonotic nature of the organism. Therefore, cheaper and easier serological tests are commonly used for assessing Brucella infection. There are several serological tests, with Rose Bengal plate test (RBPT) and enzyme-linked immunosorbent assay (ELISA) being the most commonly used serological tests for assessing Brucella infection in cattle and buffalo [4,7]. The milk ring test (MRT) is also a commonly used, cheaper, and easier test, but it is more useful for testing herd prevalence in pool milk samples (<100 L of milk), and not useful for testing dry animals and heifers. Besides, MRT may shows a false positive reaction if it is used in milk samples collected from mastitis-affected animals, animals in very early lactation (having colostrum), and those in late lactation [4,5,6,7,8].

Various studies in India have reported Brucella sero-prevalence from 0% to more than 50% in different parts of the country, but many of these studies were conducted on purposive sampling, often in a small number of animals, and therefore it is difficult to make a clear statement on the sero-prevalence of Brucella infection based on these reported studies [9]. A review in India suggests that Brucella seropositivity in the country may be around 12% [9]. In addition, the identification of risk factors relevant to sero-prevalence is important to prevent the spread of the disease, or for designing appropriate control programme. Several risk factors to brucellosis/Brucella infection have been reported [9], but many researchers have reported risk indicators (e.g., consequences of Brucella infection, such as abortion, repeat breeding, retained placenta, etc.) also as risk factors. In the present study, we have aimed to assess seropositivity and risk factors of Brucella infection in dairy animals in two of the poorest Indian states where limited studies have been conducted on brucellosis so far. The key objective of assessing risk factors is to support any brucellosis control plan/programme.

## 2. Materials and Methods

### 2.1. Data and Sampling Procedure

The cross-sectional study was conducted in the states of Assam and Bihar, India, through a primary survey of 534 dairy farming households from 52 villages in six districts over the two states, using a multi-stage sampling method. Of the total, 242 dairy farming households belonged to 24 villages of three districts of Assam, and the remaining 292 households belonged to 28 villages of three districts of Bihar. Of the total households, 46.4% (*n* = 248) were from rural areas, and the remaining 53.6% (*n* = 286) were from urban areas. The selection of the districts was guided by consultation with informed sources, especially veterinarians, in terms of the district’s potential for dairy development (low, medium, and high). Availability of primary laboratory support, as well as safety and security of the study team, were also considered during the selection of the districts. Accordingly, Kamrup (Metropolitan), Golaghat, and Baska districts were selected in Assam that represent the high, medium, and low potential districts for dairy development, respectively. Similarly, Patna, Nalanda, and Vaishali districts were selected in Bihar. In the second stage, two community development blocks (CDBs) from each district (one rural and one urban CDB) were selected. In the Patna district of Bihar, one peri-urban CDB was also selected because of the emergence of commercial dairy farming in it. For analytical purposes, the peri-urban CDB was considered as an urban CDB. Therefore, in total, seven CDBs in Bihar and six CDBs in Assam were covered. In urban areas, since the CDB and village were not found officially, the entire town/city was considered as one CDB, and different dairy clusters were considered as villages. In the third stage, from each CDB four villages were selected randomly from the list of villages within it. At the fourth stage, 10 households were selected randomly from the list of households having dairy animals (cattle and/or buffalo) in each selected village. Some key informants, such as people from local non-governmental organizations, and some leading farmers, including the village headmen, helped in preparing the list of households with dairy animals and informed the selected households in advance about the survey. All random selections were done by assigning computer-generated random numbers. Demographic characteristics of the sampled households in Assam and Bihar were described in another paper by the authors [10].

Assuming a 15% household level of the sero-prevalence of brucellosis, a 95% level of confidence, and 5% precision in the estimates, as well as using a one-sample binomial calculation, we needed at least 200 sample observations [11]. To account for a small design effect, because of clustering, we aimed at 242 households in Assam and 292 households in Bihar.

The primary survey was carried out using a systematically designed, structured questionnaire, with questions related to the location of the farm; farmers’ age, gender, education, and training completed; and farming system details, which included herd size, rearing system, breeding system, movement of animals, introduction of new animals, quarantine system followed, vaccinations given, cleanliness of farms, cleanliness of animals, breeds kept (indigenous breeds, crossbreeds, or exotic breeds, i.e., breeds not native to India), and types of floor and roofs. Different aspects of brucellosis, including knowledge about brucellosis, history of reproductive problems in the farms and animals (i.e., abortion, repeat breeding, retained placenta, vaginal discharge, carpal hygroma, and stillbirth), and brucellosis prevention practices followed in the farms (e.g., disposal of placenta, vaccination, etc.), were also included in the questionnaire. The questionnaire was pre-tested by conducting mock interviews with few farmers in the field, in order to understand if the questions were rightly framed, easily understandable, relevant, easy to respond to, and not very time-consuming. Based on the experience of the field testing, necessary changes were made before going to the actual survey. The questionnaire included both questions and physical observations by the interviewer. Global positioning system (GPS) coordinates of every surveyed households were recorded using a hand-held GPS (made by Garmin Ltd., Olathe, Kansas, United States), and the same were imported to geographic information system (GIS) platform to produce GIS maps.

Respondents provided written informed consent before the interview. The ethical approval was received from the Institutional Research Ethics Committee (IREC) of the International Livestock Research Institute (ILRI) on 21 September 2015 (no. ILRI-IREC2015-12).

### 2.2. Biological Sample Collection and Laboratory Analysis

From each selected household, blood samples were collected from a maximum of three female dairy animals of reproductive age. If there were more than three in the herd, three were randomly selected. An animal history sheet was completed for each sampled animal. Blood samples were collected in vacutainer tubes through puncturing jugular veins and were allowed to clot. Serum was separated after centrifugation and stored in cryovials at −20 °C for shipment to the Indian Council of Agricultural Research (ICAR) National Institute of Veterinary Epidemiology and Disease Informatics (NIVEDI), Bangalore, India, where serological analyses was conducted. Blood samples were collected from 829 female cattle and buffaloes from the two states. However, only 740 samples were found in good condition for analysis, of which 364 samples were from Assam and 376 samples were from Bihar. Of the total from Bihar, 354 samples belonged to cattle and the remaining 22 samples belonged to buffalo. No buffalo was found in the sampled households in Assam. The serum samples were tested by indirect ELISA (iELISA) test (produced by IDEXX Laboratories, Inc. Westbrook, Maine, United States) to assess the presence of anti-Brucella antibodies in serum samples, following the test protocol and calculation method of sample-to-positive (S/P) ratio, as recommended by the manufacturer. Following the manufacturer’s protocol, iELISA results were considered positive if the S/P ratio was found to be ≥120, questionable if the S/P ratio was found to be between 110 and 120, and negative if S/P ratio was found to be ≤110. The questionable results were considered as negative for analytical purposes. Considering the doubtful results as negative is unlikely to have biased the overall outcome of study, as both the samples belonged to Kamrup (Metropolitan), in which Brucella seropositivity was significantly higher than other two districts anyway. Furthermore, one of the suspected samples belonged to Kamrup (rural CDB), while another suspected sample belonged to Kamrup (urban CDB). This indicates that the result might not have any effect on the significance of seropositivity between rural and urban areas. Furthermore, both of the suspected samples belonged to medium-category farms. As no state-run Brucella vaccination programme was initiated in the year of survey, and no Brucella-vaccinated animal history was found in response to our question on vaccination in the survey, the possibility of a false positive reaction that might occur because of Brucella vaccination was unlikely.

### 2.3. Data Analyses

In Bihar, only one Brucella seropositive sample was found, and therefore the state was excluded from the risk analyses in this paper. Descriptive statistics was completed by producing frequency tables. Data were analysed using Stata, version-14 (STATA Corp Ltd., College Station, TX, USA). The farms were categorized into “small” (1–3 dairy animals), “medium” (4–10 dairy animals), or “large” (more than 10 dairy animals), according to the classification made by Food and Agriculture Organisation (FAO) of the United Nation (UN) in the Indian context [12]. The district classification was reorganized, as farm size was highly colinear with the districts. Large- and medium-size farms were found mainly in the Kamrup (Metropolitan) district. In the other two districts (Golaghat and Baska), only three medium size farms were found, there was no large size farm. Therefore, by combining farm size and district, the districts were reclassified into the following four groups: Kamrup (large farm), Kamrup (small farm), Golaghat, and Baska.

**Figure 1:**
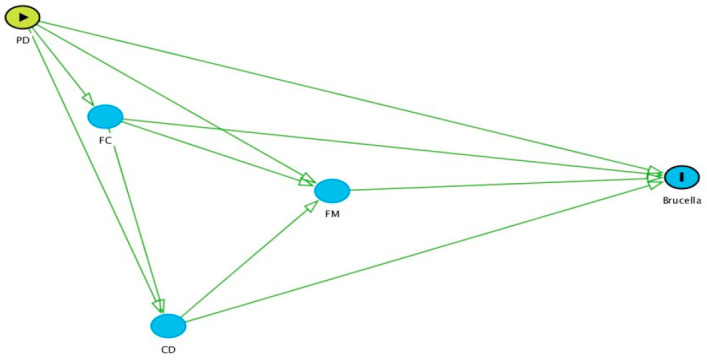
Causal diagram of potential risk factors with Brucella seropositivity. PD: producer demographics, FC: farm characteristics, CD: cattle demographics, FM: farm management. Here, arrows indicate the probability of causation of the outcome (Brucella seropositive) by different potential risk factors group.

Statistical analyses were conducted to assess risk factors for Brucella seropositivity at the animal level. Initially, univariable analyses were conducted to study the associations between Brucella seropositivity and all independent variables. A Pearson Chi-square test or Fisher’s exact test (when assumptions for the Pearson Chi-square test were not met) was employed for analysing two binary or categorical variables (nominal or ordinal), while a mean difference test (*t*-test) was employed for analysing continuous variables. Regression coefficients were obtained from simple logistic regression between the outcome variable and respective independent variables. Multivariable assessment of risk factors was carried out in a series of multilevel logistic regression models. The outcome of interest was seropositivity by ELISA at the cow level. A systematic process was followed for multivariable model building. Initially, a causal diagram was drawn (Figure 1) to show the possible relationships between groups of potential risk factors and the outcome. To simplify the diagram (and the subsequent analyses), potential risk factors were grouped into four categories: producer demographics (PD; e.g., gender, education, training availed by farmers, interaction had with veterinarians, etc.), farm characteristics (FC; e.g., location of the farms, farm size, floor type, dairy animal in contact with goat, etc.), cow demographics (CD; e.g., age, breed, etc.) and farm management (FM; e.g., animal movement, introduction of new animals, artificial insemination, use of disinfectant in cleaning the farms, etc.).

### 2.4. Criteria Followed for Selection of the Variables for Multivariable Analysis

In order to select suitable variables for model building, associations between the variables within each group of variables were assessed using the following methods:

- For all pairs of variables within a group (e.g., FC, FM, etc.) in which both variables in the pair were either continuous or dichotomous, correlations were computed;
- For all pairs of variables within a group (e.g., FC, FM, etc.) in which one variable was continuous and the other was categorical (the continuous variable was considered as the outcome and the categorical variable was converted to a set of dummy variables), *R*^2^ and a *p*-value were computed using linear regression;
- For all pairs of variables within a group (e.g., FC, FM, etc.) in which one variable was categorical and the other variable was either categorical or dichotomous, a *p*-value was computed using a Chi-square test.

Thereafter, a few selection criteria were followed as stated below for identifying suitable variables for multivariable models:

- A variable that did not have a plausible biological relationship to the outcome variable was excluded;
- A variable that did not have substantial variability (e.g., species of dairy animals) was excluded;
- A variable that had more than 10% missing values was excluded;
- If correlation between two variables was greater than 0.7 or *R*^2^ > 0.5 (as computed above), the following selection criteria were used to exclude one variable in favour of the other: (1) the variable that had a weaker association with outcome variable was excluded, (2) the variable that had higher missing value was excluded, (3) the variable for which it was relatively difficult to collect accurate data was excluded;
- A variable that had no significant association with the outcome variable (*p* > 0.3) was excluded.

### 2.5. Steps Followed for Model Building for Multivariable Analyses

After selection of the candidate variables, the following steps were followed for each multivariable analysis:

- Separate models were built for each group of variables (i.e., PD, FC, CD, and FM).
- Following the causal diagram, initially, an analysis was conducted using variables in the PD group. This was followed by the FC, CD, and FM groups in sequence. For each group, significant variables from the antecedent groups (i.e., to the left of the group of interest) were retained to see if there was any confounding effect. No variables from subsequent groups were included, as these would be intervening (intermediate) variables. For example, when analysing variables in the CD group, significant variables from the PD and FC models were retained, but all variables in the FM group were excluded.
- During analyses, variables were manually eliminated one at a time. The variable for elimination was selected based on its *p*-value, absence of evidence of confounding effects (i.e., changes in the magnitude of other coefficients), and the plausibility of its causal association with Brucella seropositivity.
- A variable was dropped from the analysis if it had strong collinearity with another variable that had more plausible association with Brucella seropositivity.
- In the final model, only significant variables (*p* < 0.1) identified through multivariable regression analysis were kept, along with the pre-selected confounders, if any. A cut point of *p* < 0.1 was chosen in light of the relatively small sample size and low prevalence of Brucella seropositivity.

The hierarchical structure of the data was accounted for by forcing the combined district/farm size variable in as a fixed effect in all models and including village and household as random effects. For each group of variables, the final model showed the total effects of the selected variables in that model. Their direct effects (after adjustment for intervening variables) was determined from the final model of the final (FM) group. Regression diagnostics were carried out on all final models. These primarily consisted of evaluating the normality and homoscedasticity of the random effects at the household and village levels and looking for outlying observations.

## 3. Results

### 3.1. Brief Profile of Farming System and Dairy Animals

With respect to the profiles of farming systems in both Bihar and Assam, we found that small farms (1–3 dairy animals) were the most predominant, both in Bihar (78.8%) and Assam (76.4%), followed by medium (4–10 dairy animals) and large farms (>10 dairy animals). The mean herd size of dairy farms in Bihar was significantly (*p* = 0.009) smaller than in Assam (Table 1). Herd size was almost uniform across the surveyed districts in Bihar, while in Assam, it largely varied among the surveyed districts. Furthermore, improved (exotic or exotic crossbreed) dairy animals were predominant in the study areas of Bihar (91.8%), while in Assam little more than half of the dairy animals in the study areas were improved, and the remaining were non-descript indigenous/indigenous breeds. In the studied households of Bihar, both cattle and buffalo were used for milk production, whereas in Assam only cattle were used for milk production. In addition, a fully stall-fed (zero-grazing) system of rearing was more common in Bihar than in Assam. Artificial insemination (AI) was found to be the most common breeding system followed in Bihar rather than in Assam.

Since our risk factor analysis focused only on Assam data, the variability of the dairy farming system under three different geographical districts of the state have been explained below (Table 2).

It was observed that farms in the Kamrup (Metropolitan) district were larger, more confined, had more improved cattle, used more AI, had more highly trained owners, and used more veterinary services and disinfectants than farms in the other two districts. Hardly any farms (only 3%) in the Kamrup (Metropolitan) district had any provision of quarantine shed or other biosecurity infrastructure. Although about 29.5% farms (Table 2) in the district reported using disinfectant, none of them used disinfectant regularly. The case was the same with regard to other biosecurity practices followed during milking, disposal of aborted materials, etc. (Supplementary Table S1).

### 3.2. Laboratory Results of Serum Samples

In total, 58 (15.9%) samples were found to be seropositive by iELISA for Brucella infection in Assam, and one (0.3%) in Bihar. The lone positive sample in Bihar belonged to the rural areas of the Nalanda district. No buffalo sample was found to be seropositive. Herd level seropositivity was 16.5% in Assam and 0.3% in Bihar. Among Brucella seropositive farms in Assam (total of 40), 23 farms had only one positive animal, 15 farms had two positive animals, and two farms had three positive animals in each. Within Assam, the highest animal level seropositivity was recorded in the Kamrup (Metropolitan) district (29.5%), followed by the Baska (4.3%) and Golaghat (2.1%) districts. Furthermore, higher Brucella seropositivity was recorded in urban areas (18.7%) than in rural areas (12.4%) of Assam, but the difference was not statistically significant.

**Figure 2:**
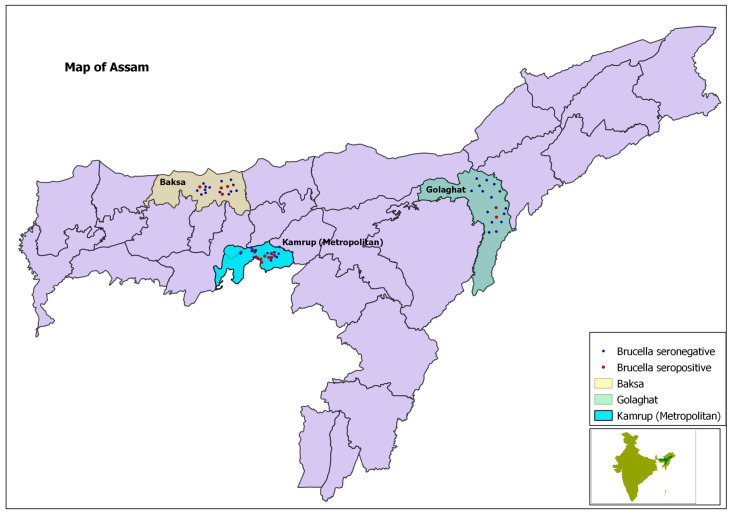
Map of Assam, showing project districts in different colours and location of surveyed households with dots.

**Figure 3:**
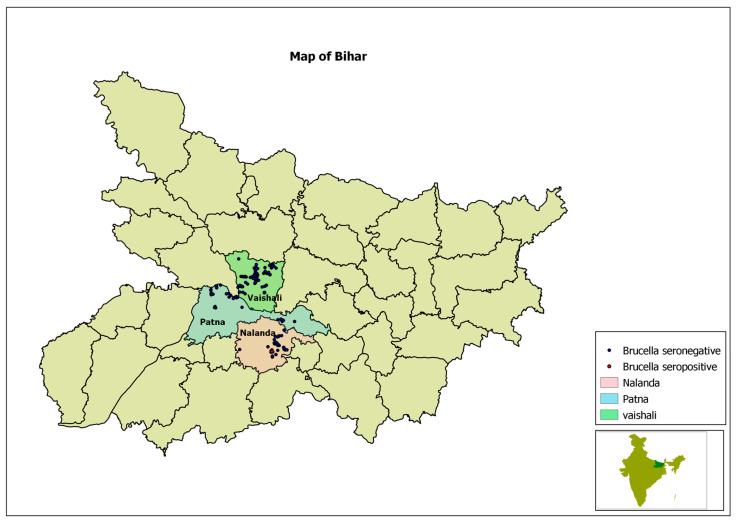
Map of Bihar, showing project districts in different colours and location of surveyed households with dots.

The locations of positive and negative households are pictorially presented in a GIS maps in Figure 2 and Figure 3.

### 3.3. Univariable Analysis

Out of 30 variables that were studied for assessing risk factors, only 15 variables were selected for multivariable analysis, using the selection criteria as described in the Methodology section. Details of the selected variables are presented in Table 3, and a complete list of all 30 variables is presented in the Supplementary Table S1. Significant (*p* ≤ 0.01) differences were observed between Brucella seropositivity and reclassified districts (Table 3). Animals belonging to the Kamrup (large farms) district were more likely to suffer from Brucella infection than the animals belonging to other three districts.

Under FC, Brucella seropositivity was found to be significantly higher in dairy animals belonging to urban areas, large-sized farms, dairy animals not in contact with goats, and those belonging to farms having concrete or other types of floors (e.g., brick floor).

Under FM, Brucella seropositivity was found to be significantly higher in cases of dairy animals that were bred through AI and belonging to the farms that introduced new animals. Contrary to the plausible biological association, Brucella seropositivity was found significantly higher in case of animals belonging to farms that did not move their animals and animals belonging to farms that used disinfectant.

Under PD, Brucella seropositivity was significantly higher in dairy animals belonging to uneducated farmers and younger farmers (20–40 years old). Contrary to the common belief, animals belonging to farmers who completed training on dairy cattle management and who had interaction with veterinarians suffered more from Brucella infection.

Under CD, significantly more animals belonging to improved breeds and animals in higher age groups suffered more from Brucella infection.

### 3.4. Multivariable Analysis of Risk Factors

The selected variables under each sub-group were used for logistic regression analysis separately within each sub-group, and the outcome of analysis are presented in Table 4.

Under the PD group, no significant association was observed between Brucella seropositivity and the age of farmers, training completed by farmers, consultation with veterinarians, and the category of education of farmers.

From the sub-group level regression analysis on FC, it was found that chances of dairy animals being Brucella seropositive in Kamrup (small farms), Baka, and Golaghat were reduced by approximately 86%, 93%, and 97%, respectively, compared to Kamrup (large farms). No significant association was observed between Brucella seropositivity and floor type or contact with goats, so both the variables were dropped during the process of regression analysis. Strong collinearity was observed between Brucella seropositivity and the farm category.

Among the variables under CD, it was found that for the increase of every year of age of dairy animal, there was approximately a 20% increase in the chance of being Brucella seropositive. There was no collinearity observed between age of animals and breed. However, breed and breeding system (i.e., AI and natural mating) were found to be highly associated.

Under the FM group, it was found that the chances of being Brucella seropositive was cut by approximately two-thirds by the adoption of AI, although this effect was only borderline significant (*p* = 0.07). No significant association was observed between Brucella seropositivity and use of disinfectants in cleaning the farms and introduction of new animals in regression analysis. Animal movement was found to be strongly collinear with the district variable.

The model, in which all the sub-models were considered together, identified the districts (to which animals belonged), age of dairy animals, and adoption of AI as the important risk factors for being Brucella seropositive (Table 5). The odds ratio was found to be almost the same with the sub-group level regression analyses, indicating that the effects mediated through intervening variables were quite weak. In all the regression analysis, the random effect of villages and households were considered. The random effects at the village and household levels were fairly small. Estimates of the intra-class correlation coefficient for each of these effects were 0.10 (village) and 0.12 (household), indicating only a low–moderate level of clustering at these levels.

## 4. Discussion

### 4.1. Sero-Prevalence

We found that dairy farming system in both Bihar and Assam had some significant differences in terms of herd size, farm category, rearing system, and breeding method, despite the fact that the small holder farming system was predominant in both states. In Assam, differences in production system among the districts might be influenced by local market demand and ease of access to fresh milk market, as about 97% of the milk in Assam is marketed through informal dairy value chain actors [13] without pasteurization. A higher concentration of large- and medium-sized farms in Kamrup (Metropolitan) districts, particularly in and around Guwahati city, the capital city of Assam where about 1.0 million people live [14], was observed, possibly to meet the demand of fresh milk in the city. No large farm was found in the other two studied districts of Assam, likely because of an absence of major urban consumption centers in both districts. In Bihar, a cooperative system of dairy production was more common (total of 1.0 million cooperative members compared to only 12,000 cooperative members in Assam) [15]; in this system, members procure milk from dairy farms located in different parts of the state and pasteurize the collected milk in dairy plants before selling to consumers. Therefore, ease of access to a fresh milk market was not a critical requirement for dairy farming in different districts of Bihar. Possibly because of that, the dairy farms were more uniformly distributed across the districts. Furthermore, every cooperative generally provides access to AI services to its farmers, which explains the more common adoption of AI in Bihar than in Assam (Table 1).

Laboratory results revealed that animal levels of seropositivity of Brucella infection in Bihar was only 0.3%, in contrast to 15.9% in Assam. No buffaloes were found to be positive in this study, but due to the low number of buffaloes included, this cannot be seen as representative. An earlier study conducted in 23 states of India reported an overall sero-prevalence of 3.5% in Bihar [16]. A few other studies have reported even higher seropositivity in Bihar (12.2% by Pandian et al. [17] and 9.3% by Kaushik et al. [18]). These findings were contrary to our study results. One possible reason could be that none of the above-mentioned studies were conducted on randomly selected samples. Lower seropositivity in Bihar could be explained by the fact that Bihar had a significantly lower mean herd size of dairy farms, higher adoption of AI, and lower mean age of dairy animals in comparison to Assam. and all three factors were identified as the important risk factors for Brucella seropositivity in our study.

On the other hand, overall seropositivity found in the present study in Assam (15.9%) agreed with previously reported estimates: 14.3% [19] and 13.84% at the animal level [20]. In Assam, our district level seropositivity was in agreement with the findings of Gogoi et al., who reported the highest seropositivity in the Kamrup (Metropolitan) district (28.6%) among nine studied districts of Assam [20]. Aulakh et al. reported significant variation of Brucella seropositivity in different districts of the Punjab state in India [21]. They reported significantly higher Brucella seropositivity in the districts where there was larger herd size with more introduction of new animals. This was mainly because in those districts, farmers used to sell Brucella-infected animals, as there was no system of screening of the animals for Brucella before selling/purchasing. In our study, we found (Table 3) that Brucella seropositivity was significantly higher (*p* = 0.006) in the farms in which new animals were introduced than those where new animals were not introduced. Our finding was also in agreement with the reports of Muma et al. and Mugizi et al., who reported that geographic location had a significant association with Brucella seropositivity [22,23]. However, our finding of Brucella seropositivity in the Kamrup (Metropolitan) district was much lower than the reported positivity of 72.5% by Lindahl et al., based on milk samples using a milk ELISA test [24]. This was the highest reported Brucella seropositivity in Assam, but we could not speculate any reason behind such higher positivity in the study.

We had also observed significantly higher Brucella seropositivity in urban areas than in rural areas. This might be because of a higher presence of large-category farms in urban areas to meet the market need of fresh milk in nearby urban centres. In rural areas, farmers possibly rear small herd of dairy animals for subsistence purpose.

### 4.2. Risk Factors

Out of 15 potential risk factors used for multivariable regression analysis, only three factors were identified as significant in multivariable regression analyses. The identified risk factors were (a) districts to which animals belong, (b) age of the animals, and (c) adoption of artificial insemination for breeding.

With regard to district, significantly higher seropositivity in Kamrup (large farms) was observed, possibly because of the dominance of large-sized farms in the district. This is in agreement with several other studies [20,23,25,26] that reported that large-sized farms are more likely to suffer from Brucella infection than small-sized farms. This higher prevalence in large-sized farms may be due to the confinement of a higher number of animals, leading to a greater chance of exposure to infected animals and aborted materials [26,27,28]. Furthermore, we have found that districts have a significant correlation with rearing system and farm size (Table 2). Larger farms mainly follow a fully stall-fed system of rearing. Several studies have suggested that fully stall-fed farms are more likely to suffer from Brucella infection than partly stall-fed farms [28,29,30], which is in corroboration with our study findings. We found that large farms introduced significantly more new animals, some of which might be from Brucella infected herds. This is in corroboration with Coelho et al., who have reported that the introduction of new animals is an important risk factor [31]. Furthermore, the majority of the large herds in Kamrup (Metropolitan) district were located in small plots of hilly areas mainly belonging to the government, and were managed by traditional, dairy-farming community migrants from the neighbouring country Nepal. These larger herds available in the district seemed to have poor biosecurity practices that might result in higher Brucella seropositivity. Infectious diseases like brucellosis had been shown earlier to be reduced by following proper biosecurity practices [32].

By employing multivariable analysis, we found that the age of the animal was an important risk factor. We found that with an increase in the age of the animals, the likelihood of being Brucella seropositive increases. This is likely because older animals get more chances of getting expose to Brucella infection than younger animals, and Brucella-infected animals might remain seropositive for a long time. Studies in India, Tanzania, Tajikistan, and Uganda have reported that older dairy animals are more likely to be seropositive than younger animals [21,23,26,33]. However, some other studies have reported that younger animals are more likely to be Brucella seropositive than older animals [34,35].

Furthermore, we found that dairy animals bred through AI were less likely to be Brucella seropositive than animals bred through natural mating. This might be because AI is a well-established biosecurity measure, as no movement of bulls takes place and no physical contact between male and female dairy animals happens that eliminate the chances of transmitting Brucella infection. Furthermore, in village conditions, community bulls are more commonly used by farmers for breeding. If such a bull suffers from brucellosis, that could easily transmit the disease from one animal to the other. This is in corroboration with few other studies that have observed higher seropositivity in the animals bred through natural mating than AI [34,36]. Contrary to our findings, some other researchers have not found any significant association between Brucella seropositivity and AI [37].

Interestingly, by employing univariable analysis, we found that there was a greater likelihood of being Brucella seropositive if the animals belonged to farmers who completed training on dairy cattle management, or who had consulted with veterinarians. Furthermore, we found that there was a greater likelihood of being Brucella seropositive if the animals were not moved and if the farms used disinfectants in cleaning the farms. These implausible associations were perhaps the results of confounding effect of district, and once the district was controlled by accounting for farm size, the variables became non-significant in multivariable analysis.

### 4.3. Comments on Study Design

The multi-stage, random sampling method helped generate an unbiased selection of villages, households, and animals. While it might have been desirable to also select districts randomly, this was not done because of the requirement of minimum laboratory infrastructure for initial processing of the samples in the districts. Conducting the study in different districts, as well as rural and urban settings, has given a more comprehensive idea about the seroprevalence and risk factors of Brucella infection.

## 5. Conclusions

The present study suggests that Brucella infection may significantly vary between states or districts based on different factors. Our seropositivity result in Assam was in confirmation with the earlier findings, but the result of seropositivity in Bihar was much lower than expected. For proper assessment of Brucella seropositivity, larger size samples collected through a random sampling method is critical. Chances of being Brucella seropositive were lower in dairy animals in districts with smaller-size herds than districts having larger size herds. Furthermore, younger dairy animals are less likely to be Brucella seropositive than older animas, and animals bred through AI are less likely to be Brucella seropositive than those bred through natural mating. All three identified risk factors might partly explain the reason behind lower Brucella seropositivity in Bihar. Therefore, in any future brucellosis control program, appropriate care should be taken to reduce the risk of Brucella infection by addressing the identified risk factors by way of proper communication and capacity building of the dairy farming community.

## Figures and Tables

**Table 1:** Profile of the dairy animals among the surveyed households, with *p*-values of differences between Bihar and Assam, India.

Variables	Particulars	Bihar	Assam	Total	*p*-Value
Mean Herd Size per Farm (± standard error (SE))		2.8 ± 0.2	4.1 ± 0.4	3.4 ± 0.2	0.009
Breed of the animals surveyed	High-producing (exotic or crossbred)	345/376 (91.8) *	186/364 (51.1)	531/740 (71.8)	<0.001
	Non-descript indigenous	31/376 (8.2)	178/364 (48.9)	209/740 (28.2)	
Species of the animals	Cattle	354/376 (94.1)	364/364 (100.0)	718/740 (97.0)	<0.001
	Buffalo	22/376 (5.9)	0/364	22/740 (2.9)	
Mean age of animals surveyed (± SE)		4.7 ± 0.1	6.2 ± 0.1	5.4 ± 0.1	<0.001
Breeding method followed by households	Followed artificial insemination (AI)	266/292 (91.1)	128/242 (52.9)	394/534 (73.8)	<0.001

* Figures in the parenthesis indicate percentage of the corresponding total.

**Table 2:** District-wise profile of the dairy animals, with *p*-values of differences between the three districts in Assam.

Variables	Particulars	Kamrup (Metropolitan)	Golaghat	Baska	*p*-Value
Mean herd size of dairy animals	Per farm	8.8 ± 1.1	1.7 ± 0.1	1.6 ± 0.1	0.01
Rearing system	Fully stall-fed	141/178 (79.2) *	9/105 (8.6)	6/94 (6.4)	<0.001
	Partly stall-fed	37/178 (20.8)	96/105 (91.4)	88/94 (93.6)	
Breed	Improved	148/176 (84.1)	18/96 (18.7)	20/92 (21.7)	<0.001
	Non-descript	28/176 (15.9)	78/96 (81.2)	72/92 (78.3)	
Adoption of AI	Yes	152/178 (85.4)	45/105 (42.8)	34/94 (36.2)	<0.001
Animal movement	Yes	39/178 (21.9)	96/105 (91.4)	90/94 (95.7)	<0.001
New animal introduced	Yes	71/178 (39.9)	6/105 (5.7)	4/94 (4.2)	<0.001
Animals belonging to trained farmers	Yes	41/178 (23.0)	5/105 (4.8)	5/94 (5.3)	<0.001
Animals belonging to farmers who had consultation with veterinarian	Yes	158/178 (88.8)	77/105 (73.3)	70/94 (74.50)	0.001
Use of disinfectants in cleaning the farms	Used	52/176 (29.5)	2/96 (2.1)	4/92 (4.3)	<0.001

* Figure in the parenthesis indicates percentage of the corresponding total.

**Table 3:** Selected variables for multivariable model building with their characteristics.

Variables	Description of the Variables	Sero-Positive/Total (%)	Coefficient of Unconditional Association with Brucella Sero-Positivity	*p*-Value of Unconditional Association with Brucella Sero-Positivity	Missing Value	Kept for Multivariable Model
**Outcome**						
ELISA results of Brucella infection	Positive	58			13	
	Negative	306				
**Identifiers**						
Districts	Kamrup (large farms)	49/135 (18.9)	Ref. *	<0.001	0	Yes
	Kamrup (small farms)	3/41 (7.3)	−2.19			
	Golaghat	2/96 (2.1)	−2.77			
	Baska	4/92 (4.3)	−3.55			
**Farm Characteristics (FC)**						
Location of the farm in rural or urban areas	Rural CDB	20/161 (12.4)	Ref.	0.160	0	Yes
	Urban CDB	38/203 (18.7)	0.43			
Category of farms	Small (1–3 dairy animals),	8/223 (3.6)	Ref.	<0.001	0	Yes
	Medium (4–10 dairy animals)	19/81 (23.4)	2.19			
	Large (>10 dairy animals)	31/60 (51.7)	3.58			
Dairy animals in contact with goats	Yes	9/106 (8.5)	−1.19	0.030	0	Yes
	No	49/258 (19.0)	Ref.			
Type of floor	Concrete	20/67 (29.8)	Ref.	<0.001	0	Yes
	Earthen	9/202 (4.4)	−2.42			
	Others	29/95 (30.5)	0.10			
**Farm Management (FM)**						
Adoption of AI	Yes	46/225 (20.4)	1.16	0.020	0	Yes
	No	12/139 (8.6)	Ref.			
Introduction of new animals	Introduced	22/79 (27.8)	1.54	0.006	0	Yes
	Not introduced	36/285 (12.6)	Ref.			
Animal movement	Animal moved	9/213 (4.2)	−2.67	<0.001	0	Yes
	Not moved	49/151 (32.4)	Ref.			
Use of disinfectant in cleaning farms	Used disinfectant	35/160 (21.9)	1.06	0.020	0	Yes
	Not used disinfectant	23/204 (11.3)	Ref.			
**Producer Demographics (PD)**						
Education of farmers	No education	17/66 (25.7)	Ref.	0.14	0	Yes
	Class I–V	11/49 (22.4)	−0.15			
	Class VI–X	18/149 (12.1)	−1.16			
	Class XI and above	12/100 (12.0)	−1.13			
Age of farmers	20–40 years	21/89 (23.6)	Ref.	0.10	0	Yes
	41–60 years	26/191 (13.6)	−1.15			
	60 years and above	11/84 (13.1)	−1.15			
Training completed by farmers	Completed	15/50 (30.0)	1.52	0.02	0	Yes
	Not completed	43/314 (13.7)	Ref.			
Interaction had with the veterinarians	Had interaction	55/297 (18.5)	1.85	0.005	0	Yes
	No interaction	3/67 (4.5)	Ref.			
**Cow Demographics (CD)**						
Breed of animal	Non-descript indigenous	7/178 (3.9)	Ref.	<0.001	13	Yes
	Improved/CB/pure	51/186 (27.4)	2.66			
Age of animals	With Brucella seropositive	6.83 ± 0.33		0.03	13	Yes
	With Brucella sero-negative	6.09 ± 0.13				

Note: Last one year means last 12 months from the date of survey. ELISA: enzyme-linked immunosorbent assay; CDB: community development block. * Reference.

**Table 4:** Results of the four sub-models used in the analysis.

	Odds Ratio	Standard Error	*p*-Value	95% Confidence Interval
**Producer Demographics (PD)**				
District				
Kamrup (large farms)	Ref. *			
Kamrup (small farms)	0.14	0.10	0.007	0.030–0.590
Baska	0.07	0.04	<0.001	0.020–0.250
Golaghat	0.03	0.03	<0.001	0.006–0.160
**Cow Demographics (CD)**				
Age of the dairy animals, in years	1.23	0.10	0.008	1.050–1.440
**Farm Management (FM)**				
Artificial insemination adopted	0.33	0.20	0.070	0.100–1.100

* Reference.

**Table 5:** Models with the identified risk factors from all sub-group models.

Variables	Odds Ratio	Standard Error	*p*-Value	95% Confidence Interval
District				
Kamrup (large farms)	Ref. *			
Kamrup (small farms)	0.11	0.09	0.007	0.020–0.540
Baska	0.03	0.02	<0.001	0.005–0.150
Golaghat	0.01	0.01	<0.001	0.002–0.100
Age of dairy animals, in years	1.24	0.10	0.007	1.060–1.440
Artificial insemination adopted	0.33	0.20	0.072	0.100–1.100
Random effect of village	0.40	0.49		
Random effect of household	0.51	0.69		

* Reference.

## Data Availability

Data will be made available from the authors upon request.

## Supplementary Materials

The following are available online at https://www.mdpi.com/article/10.3390/microorganisms9040783/s1, Table S1: List of all the variables studied to assess risk factors of Brucella infection with their particulars, and our decision on using it for multivariable analysis.
