# Supplementary material for: Seroprevalence and Risk Factors of Brucella Infection in Dairy Animals in Urban and Rural Areas of Bihar and Assam, India
Source: Microorganisms. 2021 Apr 9;9(4):783. doi: 10.3390/microorganisms9040783 (PMC8070207; doi:10.3390/microorganisms9040783)
Supplement: Supplementary file 1 [file microorganisms-09-00783-s001.pdf]

*Supplementary materials:*

**Table S1: List of all the variables studied to assess risk factors of Brucella-infection with their particulars and our decision on using it for multivariable analysis**

| Variables | Description of the variables | Sero- positive/ total (%) | Coefficient of unconditional association with Brucella sero-positivity | p-value of association | Missing value | Kept for multivariable model |
|---|---|---|---|---|---|---|
| **Outcome** | | | | | | |
| ELISA results of Brucella-infection | Positive | 58 | | | 13 | |
| | Negative | 306 | | | | |
| **Identifiers** | | | | | | |
| Districts | Kamrup (large farms) | 49/135 (18.9) | Ref. | <0.001 | 0 | Yes |
| | Kamrup (small farms) | 3/41(7.3) | -2.19 | | | |
| | Golaghat | 2/96(2.1) | -2.77 | | | |
| | Baska | 4/92(4.3) | -3.55 | | | |
| **Farm Characteristics (FC)** | | | | | | |
| Location of the farm in rural or urban areas | Rural CDB | 20/161(12.4) | Ref. | 0.16 | 0 | Yes |
| | Urban CDB | 38/203(18.7) | 0.43 | | | |
| Category of farms | Small (1-3 dairy animal), | 8/223(3.6) | Ref. | <0.001 | 0 | Yes |
| | Medium (4-10 dairy animals) | 19/81(23.4) | 2.19 | | | |
| | Large (>10 dairy animals) | 31/60(51.7) | 3.58 | | | |
| Dairy animals in contact with goat | Yes | 9/106(8.5) | -1.19 | 0.03 | 0 | Yes |
| | No | 49/258(19.0) | Ref. | | | |
| Type of floor | Concrete | 20/67(29.8) | Ref. | <0.001 | 0 | Yes |
| | Earthen | 9/202(4.4) | -2.42 | | | |
| | Others | 29/95(30.5) | 0.10 | | | |
| System of rearing | Fully stallfed | 50/155(32.2) | 2.48 | <0.001 | 0 | No |
| | Partly stallfed | 8/209(3.8) | Ref. | | | |
| Dairy animal in contact with pig | Yes | 2/14(14.3) | -0.13 | 0.86 | 0 | No |
| | No | 56/350(16.0) | Ref. | | | |
| Dairy animal in contact with dog | Yes | 33/216(15.3) | -0.12 | 0.68 | 0 | No |
| | No | 25/148(16.9) | Ref | | | |
| Dairy animal in contact with wild animal | Yes | 25/159(15.7) | -0.03 | 0.92 | 0 | No |
| | No | 33/205(16.1) | Ref. | | | |
| Type of roof | Thatch | 9/46(19.6) | 0.19 | 0.49 | 77 | No |
| | Corrugated Tin/ asbestos | 28/233(12.0) | 0.12 | | | |

| | | | | | | |
|---|---|---|---|---|---|---|
| | Others | 2/18(11.1) | Ref. | | | |
| Source of introduction | Known source | 19/67(28.3) | 0.17 | 0.80 | 285 | No |
| | Unknown source | 3/12(25.0) | Ref. | | | |
| **Farm Management (FM)** | | | | | | |
| Adoption of AI | Yes | 46/225(20.4) | 1.16 | 0.02 | 0 | Yes |
| | No | 12/139(8.6) | Ref. | | | |
| Introduction of new animals | Introduced | 22/79(27.8) | 1.54 | 0.006 | 0 | Yes |
| | Not introduced | 36/285(12.6) | Ref. | | | |
| Movement of animal | Animal moved | 9/213(4.2) | -2.67 | <0.001 | 0 | Yes |
| | Not moved | 49/151(32.4) | Ref | | | |
| Use of disinfectant in cleaning farms | Used disinfectant, | 35/160(21.9) | 1.06 | 0.02 | 0 | Yes |
| | Not used disinfectant | 23/204(11.3) | Ref | | | |
| Quarantine of newly purchased animal | Yes | 0/9 | 0 | 0 | 0 | No |
| | No | 58/355(16.3) | | | | |
| Purchase of sick animal | Yes | 4/18(22.2) | 0.43 | 0.47 | 0 | No |
| | No | 54/346(15.6) | Ref | | | |
| Purchase of weak but cheap animal | Yes | 3/10(30.0) | 0.84 | 0.26 | 0 | No |
| | No | 55/354(15.5) | Ref. | | | |
| Vaccination (against any disease) followed | Yes | 46/150(30.7) | 2.01 | <0.001 | 0 | No |
| | No | 12/214(5.6) | Ref. | | | |
| Cleanliness of animal | Very clean | 20/118(16.9) | Ref. | 0.73 | 0 | No |
| | Clean | 38/244(15.6) | -0.10 | | | |
| | Dirty | 0/2 | 0 | | | |
| Aborted material buried | Yes | 28/70(40.0) | 1.20 | 0.23 | 288 | No |
| | No | 1/6(16.7) | Ref. | | | |
| **Producers Demographic (PD)** | | | | | | |
| Education of farmers | No education | 17/66(25.7) | Ref. | 0.14 | 0 | Yes |
| | Class I-V | 11/49(22.4) | -0.15 | | | |
| | Class VI-X | 18/149(12.1) | -1.16 | | | |
| | Class XI and above | 12/100(12.0) | -1.13 | | | |
| Age of farmers | 20-40 years | 21/89(23.6) | Ref. | 0.10 | 0 | Yes |
| | 41-60 years | 26/191(13.6) | -1.15 | | | |
| | 60 years and above | 11/84(13.1) | -1.15 | | | |
| Training availed by farmers | Availed | 15/50 (30.0) | 1.52 | 0.02 | 0 | Yes |
| | Not availed | 43/314(13.7) | Ref. | | | |
| Interaction had with the veterinarians | Had interaction | 55/297(18.5) | 1.85 | 0.005 | 0 | Yes |
| | No interaction | 3/67(4.5) | Ref. | | | |
| Gender of farmer | Male | 58/351(16.5) | 0 | 0 | 0 | No |
| | Female | 0/13 | | | | |

| Cow Demographic (CD) | | | | | | |
|---|---|---|---|---|---|---|
| Breed of animal | Non-descript indigenous | 7/178(3.9) | Ref. | <0.001 | 13 | Yes |
| | Improved/CB/pure | 51/186(27.4) | 2.66 | | | |
| Age of animals | With Brucella sero-positive | 6.83±0.33 | 0.12 | 0.03 | 13 | Yes |
| | With Brucella sero-negative | 6.09±0.13 | Ref. | | | |
| No. of lactation | With Brucella sero-positive | 3.24±0.23 | 0.23 | 0.009 | 0 | No |
| | With Brucella sero-negative | 2.64±0.09 | Ref. | | | |
| Species of animal | Cattle | 58/364 | 0 | 0 | 0 | No |
| | Buffalo | 0 | | | | |
